# Case report: Isolated tubular basement membrane deposits in a patient with systemic lupus erythematosus - A diagnostic challenge

**DOI:** 10.3389/fmed.2022.958615

**Published:** 2022-09-16

**Authors:** Orlando Vieira Gomes, Leonardo Fernandes e Santana, Rita Marina Soares de Castro Duarte, Mateus de Sousa Rodrigues, Jandir Mendonça Nicacio, Dyego José de Araújo Brito, Monique Pereira Rêgo Muniz, Natalino Salgado-Filho, Precil Diego Miranda de Menezes Neves, Gyl Eanes Barros Silva

**Affiliations:** ^1^University Hospital, Federal University of Vale Do São Francisco (HU-UNIVASF), Petrolina, Brazil; ^2^University Hospital, Federal University of Maranhão (HU-UFMA), São Luís, Brazil; ^3^Nephrology Division, Medical School, University of São Paulo (FM-USP), São Paulo, Brazil

**Keywords:** systemic lupus erythematosus, lupus nephritis, tubulointerstitial nephritis, pathology, treatment

## Abstract

Lupus nephritis is one of the most serious and frequent manifestations of systemic lupus erythematosus. It usually presents in the first years of the disease, which suspicion should be raised in cases of elevated serum creatinine, presence of proteinuria above 500 mg/day or active urinary sediment, in the absence of other apparent causes such as urinary tract infection and use of nephrotoxic drugs. In most cases, it affects the glomerulus, and its presentation is rare in the form of isolated tubulo-interstitial disease. In this report, we describe a case of lupus nephritis diagnosed after 2 years of illness, in the form of atypical isolated tubular disease, characterized by massive deposits in the tubular basement membrane. Clinically, there were altered renal function, subnephrotic proteinuria, and evolution to a complete clinical response after immunosuppressive treatment.

## Introduction

Tubulointerstitial kidney disease (TKD), which includes alterations in the tubular epithelium, atrophy, hypertrophy, and interstitial fibrosis, is a well-recognized finding in lupus nephritis, occurring in 66% of all renal biopsies of patients with systemic lupus erythematosus (SLE) (1). In the majority of cases, TKD is associated with severe glomerular lesions, and it may be found in all classes of lupus nephritis, especially in the proliferative ones, such as class IV (2).

There exist, however, few reports of isolated or predominant tubulointerstitial involvement in the presence of minimal glomerular alterations associated with SLE, which is known as predominant tubulointerstitial lupus nephritis (3–5).

This report describes the case of a female patient with isolated tubulointerstitial lupus nephritis, which was diagnosed 2 years after diagnosis of SLE, based on clinical and serological criteria. The patient had altered renal function and subnephrotic proteinuria.

## Case description

A 39-year-old Black female patient had, 2 years prior, been diagnosed with SLE according to the Systemic Lupus International Collaborating Clinics (SLICC) criteria (6), the following of which she fulfilled: antinuclear antibody (ANA), synovitis in 2 or more joints, anti-dsDNA, malar rash, and leucopenia. During outpatient follow-up for SLE, she denied hospitalizations or clinical complications since the last visit. She complained of fatigue on alternating days and non-restorative sleep. She was taking prednisone 20 mg/day, hydroxychloroquine 400 mg/day, and CaCO_3_ + vitamin D3 (500 mg + 400 IU/day). Upon physical examination, she presented the following: good overall condition, anicteric, afebrile, arterial pressure of 120/80 mmHg, no alterations in the cardiovascular or respiratory system, flaccid and globose abdomen without pain on superficial and deep palpation, well-perfused extremities, and no edemas. There were no alterations on physical exam related to extra-renal lupus disease activity. She brought the routine laboratory exams requested for follow-up for SLE, which are displayed in Table 1. Due to evidence of altered renal function (estimated glomerular filtration rate of 45.8 ml/min/1.73 m^2^ as calculated by the CKD-EPI equation), subnephrotic proteinuria, and active urinary sediment, which is compatible with proliferative glomerulonephritis, renal biopsy was indicated.

**Table 1:** Laboratory test.

**Laboratory Test**	**Normal range**	**Pre kidney biopsy**	**At 12 months of treatment**	**At 24 months of treatment**
Hemoglobin (g/dL)	13.5–18	11.1	13.2	13.2
Hematocrit (%)	40–54	34.2	35.0	36.0
Leukocytes (per mm3)	4.000–10.000	2.760	2.090	2.610
Lymphocytes (per mm3)	1.400–3.150	883	929	929
Platelets (per mm3)	150.000–450.000	183.000	170.000	216.000
Urea (mg/dL)	16.6–48.5	21	34.6	22
Creatinine (mg/dL)	0.5–1.2	1.44	1.0	0.99
24 h-proteinuria (mg)	<150	521	92	195
Serum potassium (mmol/L)	3.5–5.5	3.7	-	-
Urine culture	Negative	Negative		
**Urine analysis**				
Glucose	Absent	Absent		
pH	5–6.5	6.0		
Red blood cells (per high-power field)	0–3	4–7	Absent	Absent
Leucocytes (per high-power field)	0–5	0–3	6	Rare
Protein	Negative	Traces	Negative	Negative
ANA - HEp-2	Negative	1:320	1:320	1:320
Anti-double-stranded DNA	Negative	1:40	Negative	Negative
ESR (mm/1^st^ h)	<20	32	32	32
CRP (mg/L)	<6.5	6	6	6
C3 (mg/dL)	90–180	100	101	112
C4 (mg/dL)	19–52	26	25	24
Lupus anticoagulant	Negative	Negative	-	-
Anticardiolipin (IgM/IgG)	Negative	Negative	-	-
Anti-beta-2-glycoprotein I (IgM/IgG)	Negative	Negative	-	-
VDRL	Negative	Negative	-	-
Anti-HIV	Negative	Negative	-	-
Anti-HCV	Negative	Negative	-	-
HBsAg	Negative	Negative	-	-

ANA, antinuclear antibodies; CRP, C-reactive protein; ESR, erythrocyte sedimentation rate; HEp-2; VDRL, Venereal Disease Research Laboratory.

**Figure 1:**
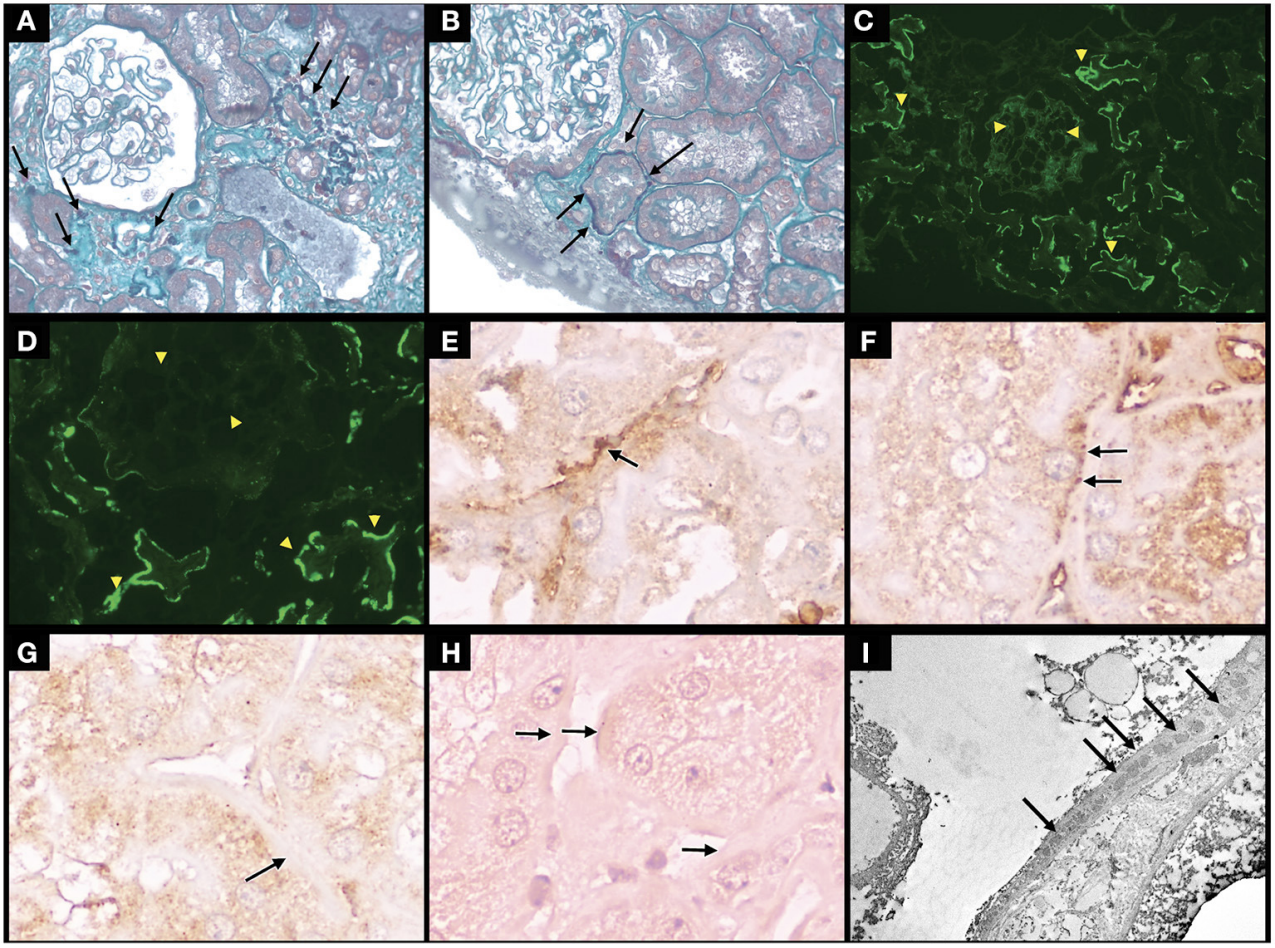
Massive immune complex depositions on the tubular basement membrane (TBM), fixing IgG and C1q, without active glomerulopathy, with mild tubulointerstitial repercussions. Immunohistochemistry for IgG1, IgG2, IgG3, IgG4, and electron microscopy study in tubular basement membrane. **(A)** Optical microscopy (OM) - Fuchsinophilic deposits in the TBM (arrows) of atrophic tubules next to normal glomerulus (Masson's trichrome stain, original magnification × 400); **(B)** OM - Fuchsinophilic deposits in the TBM (arrows) of normal tubules next to normal glomerulus (Masson's trichrome stain, original magnification × 400); **(C)** Immunofluorescence (IF) - Positivity with IgG antiserum in the TBM of tubules next to glomerulus with mild positivity (original magnification × 200); **(D)** IF - Positivity with C1q antiserum in the TBM of tubules next to negative glomerulus (original magnification x400); **(E)** TBM positive staining by immunohistochemistry for IgG1; **(F)** TBM positive staining by immunohistochemistry for IgG2; **(G)** TBM positive staining by immunohistochemistry for IgG3; **(H)** TBM positive staining by immunohistochemistry for IgG4. **(I)** Transmission electron microscopy of the paraffin-embedded tissue shows immune complex deposits (arrow) in TBM. **(E–H)**: barr = 10 μm. **(I)**: barr = 0.5 μm.

**Figure 2:**
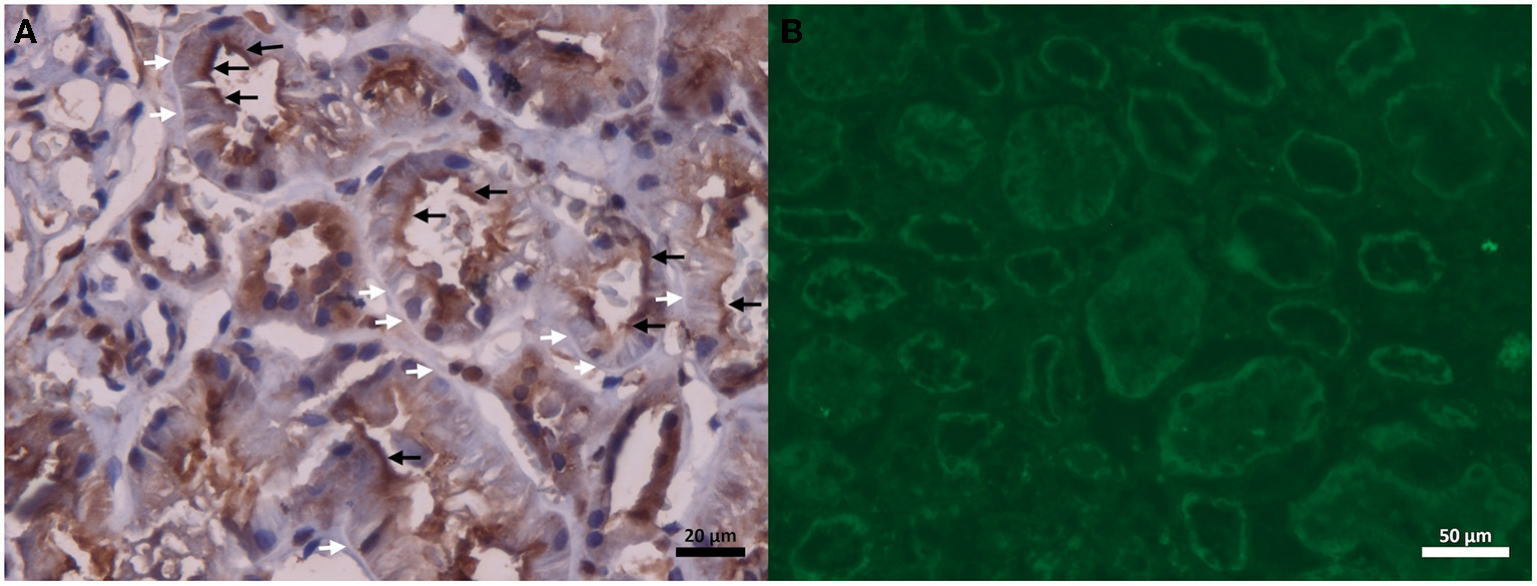
**(A)** The kidney biopsy shows normal staining for cubilin using a polyclonal antibody along the apical membrane of the tubular epithelium (black arrows), but tubular basement membrane staining was not detected (white arrow). **(B)** ABBA negative serum stains in proximal tubular epithelial brush border using a normal human kidney tissue (indirect immunofluorescence).

The renal biopsy at light microscopy revealed that 3 of the 13 glomeruli were globally sclerotic, and the remaining were normal. No abnormalities were seen in the vascular compartment. There were no signs of interstitial inflammation. Mild foci of interstitial fibrosis and tubular atrophy were detected; however, a remarkable finding of granular fuchsinophilic hyaline deposits in the tubular basement membrane (TBM) was observed (Figures 1A,B). In relation to immunofluorescence microscopy, the sample contained 6 glomeruli in conditions for analysis, and massive deposits of IgG, C3, C1q, kappa, and lambda were observed in the TBM, in addition to deposits of IgG with mild positivity in the mesangium of the adjacent glomerulus (Figures 1C,D). Immunohistochemical studies revealed predominant deposition of IgG1 and IgG2, but not IgG3 and IgG4 in the TBM (Figures 1E–H). The kidney biopsy showed normal staining for cubilin using a polyclonal antibody along the apical membrane of the tubular epithelium, but tubular basement membrane staining was not detected (Figure 2A). The patient had serum IgG anti-brush border antibody (ABBA) non-reactive to the proximal tubular brush border on sections of normal human kidney (Figure 2B).

**Figure 3:**
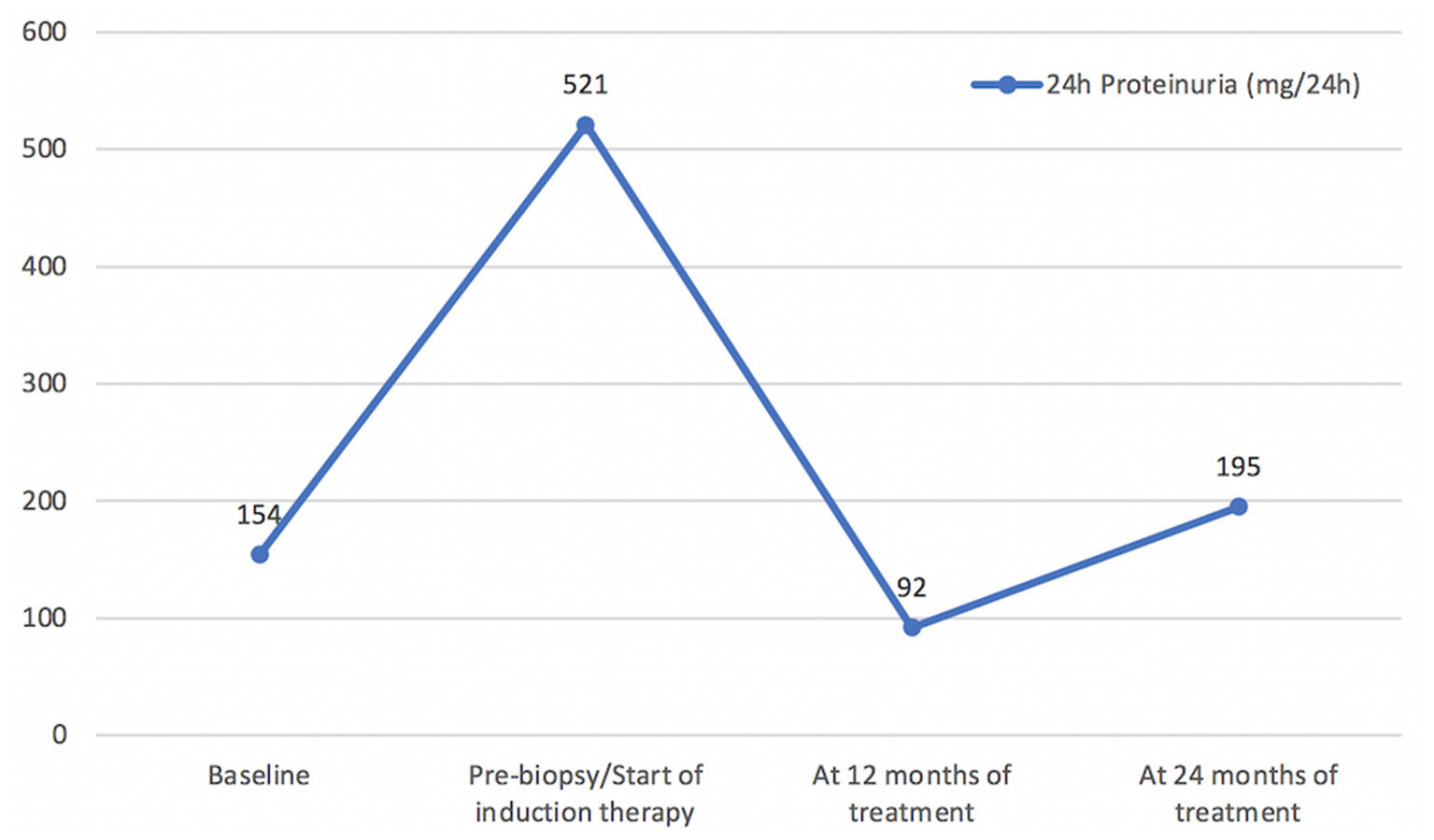
Proteinuria (mg/24 h) Timeline.

**Figure 4:**
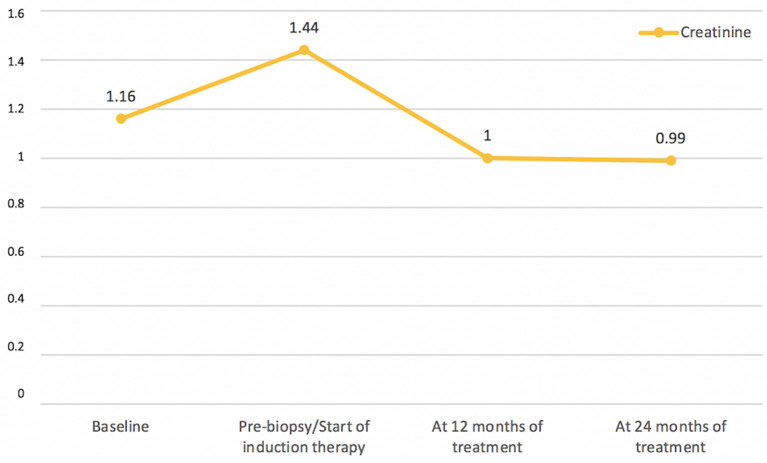
Creatinine (mg/dL) Timeline.

In the setting of an autoimmune renal tubulointerstitial disease, a screening for Sjogren's syndrome (SS) was performed, which resulted on positive Anti-SSA and negative Anti-SSB. Despite the presence of SS-A antibodies, diagnosis of SS was ruled out because the patient had no symptoms of dry mouth or eyes, and the biopsy of the minor salivary glands was normal. In post-biopsy outpatient consultation, the patient did not have any new complaints or abnormalities on physical examination. Induction therapy with mycophenolate mofetil (MMF) 3 g/day was initiated and prednisone dose was titrated to 60 mg/day due to the association of histological findings and impaired renal function, with follow-ups scheduled to evaluate clinical/laboratory response and to evaluate corticoid tapering. Induction treatment lasted 6 months, and maintenance treatment was continued with MMF 2 g/day. At 12 months, the patient showed complete remission of proteinuria (Figure 3) and improved renal function (Figure 4). During the period of treatment, the patient did not have flares of the disease, and did not require hospitalization.

## Discussion

SLE is an autoimmune disease that involves the production of autoantibodies, with the formation of immune complexes and the potential involvement of any organ. Given that it is a heterogeneous disease, its classification criteria have been adapted over time, to the extent that its pathophysiological basis is better understood (7). In this case, the SLICC 2012 had been used, because the diagnosis had been made 2 years prior, at which time those criteria were the most current and the most sensitive. At the present moment, however, new classification criteria are available to us, namely, those elaborated in conjunction by the American College of Rheumatology and the European League Against Rheumatism (ACR/EULAR), which were published in 2019 and which have been duly validated. Comparative analyses describe the compatibility of the new criteria with the older ones, maintaining good sensitivity and specificity (8). When applying the ACR/EULAR 2019 criteria to the patient in this case, we obtain the following results: ANA > 1:80 with the HEp-2 method (1:320 nuclear dot), which fulfills the opening criterion, in association with leucopenia (3 points), acute cutaneous lupus (6 points), joint involvement (6 points), proteinuria > 0.5 g/24 h (4 points), and anti-dsDNA (6 points); thus totaling 25 points.

Lupus nephritis, which is an important determinant of morbidity and mortality, is observed in up to 50% of patients with SLE, with progression to end-stage kidney disease in up to 10% to 30% of cases. It should be suspected whenever there is active urinary sediment, proteinuria > 500 mg/24 h, or impaired renal function evidenced by increased serum creatinine. The most common of these abnormalities is proteinuria, which is present in virtually all cases. Classically, lupus nephritis manifests in the form of glomerulopathy, according to the International Society of Nephrology and the Renal Pathology Society (ISN/RPS 2003) classification, which varies between classes I (minimal mesangial), II (mesangial proliferative), III (focal proliferative), IV (diffuse proliferative), V (membranous), and VI (sclerosing) (9).

TKD in patients with lupus manifests with decreased glomerular filtration rate, whether or not it is accompanied by signs of tubular dysfunction, such as renal tubular acidosis, hyperkalemia, or secondary hyperaldosteronism. The urine sediment may be completely normal, or it may present non-glomerular hematuria and pyuria (5). TKD is a potential finding that can occur concomitantly with any class of lupus nephritis, with higher incidences in classes III/IV and lower incidences in classes I/II (10). The tubulointerstitial compartment is rarely affected in an isolated manner, and the presence of massive immunoglobulin deposits in the tubule interstitium is even rarer in the absence of prominent structural changes on optical microscopy, in a manner analogous to the reported case (3–5). Moreover, the pathophysiology of TKD is still little understood, but it appears to involve the formation of immune complexes *in situ*, regardless of the degree of severity of the systemic autoimmunity state (10). Currently, due to the scarce number of cases described, there is no specific therapy, and there are no clinical trials evaluating the treatment of TKD in these patients. Most of the cases described have been managed with systemic corticotherapy alone, with no addition of other immunossupressant agent (4, 5, 11).

We have reported the case of a female patient who had proteinuria above 500 mg/24 h, urinary sediment abnormality (mild hematuria), and elevated serum creatinine, making it mandatory to investigate lupus nephritis by renal biopsy. In the present case, there was no evidence of glucosuria or urinary pH abnormalities, which makes the hypothesis of Fanconi syndrome less likely. The serum potassium was within the normal range. The evidence of massive immune complex deposition in the TBM, fixing IgG, C3, kappa, lambda, and C1q, as well as mild mesangial immune deposits of IgG without mesangial hypercellularity (class I: minimal mesangial lupus nephritis), with mild tubulointerstitial repercussion, in association with the patient's clinical and laboratory conditions, left no doubt as to the diagnosis of TKD secondary to SLE. Using immunohistochemical evaluations, IgG1 dominance/codominance with concomitant IgG2 but absent IgG3 and IgG4 staining favors an underlying autoimmune disease. We emphasize that this is the only case, of which we are aware, where there was a marked divergence between the findings of immunofluorescence (massive immune complex deposition in the TBM) and optical microscopy (minimal structural alterations), in TKD. We also highlight that these findings, in the absence of criteria that fulfill definitive diagnosis of SLE, should indicate suspicion of other nosological entities, such as the presence of ABBA disease, IgG4 disease, hypocomplementemic tubulopathy, and Sjogren's syndrome. In all of these pathologies, the characteristic finding of mononuclear inflammatory infiltrate, which is very scarce in the present case, is worthy of attention (2).

Although ABBA disease has been reported in the setting of lupus nephritis (12), in our patient that diagnosis would not be suitable. IgG staining (mostly IgG4) in the apical surface of the tubular epithelium and the basolateral basement membrane is a typical finding in ABBA disease. On light microscopy, ABBA disease shows variable but often severe interstitial fibrosis and tubular atrophy, as well as interstitial inflammation with focal tubulitis (13). In addition, no reported case of ABBA disease has shown positivity with C1q in the tubular basement membrane. On the other hand, C1q tubular basement membrane deposits are a common finding in lupus. Additionally, the findings of massive deposits of IgG (only IgG1 and IgG2), C3, kappa, and lambda of IgG1 and IgG2 appear similar to a new form of ABBA disease due to cubilin and amnionless antibodies (14). However, in our patient, there was complete lack of cubilin staining in the tubular basement membrane. Indirect immunofluorescence was also performed using the patient's serum and kidney tissue from a control, which ruled out ABBA disease. A detailed medical history revealed no evidence of antibiotic use or other potentially nephrotoxic medications.

## Patient perspective

TKD associated with SLE, in an isolated manner, is extremely rare, and it worsens renal prognosis (5, 10). We have reported the case of a patient in whom the isolated involvement of the tubular basement membrane without associated interstitial lesion is remarkable. In a manner analogous to the reported case, TKD does not usually occur with consumption of serum complement or high titers of anti-dsDNA (10). With respect to clinical management, in this case, remission induction therapy was carried out with MMF 3 g/day for 6 months, with subsequent dose reduction to 2 g/day for maintenance therapy. The patient recovered renal function and expressively reduced proteinuria, without any new flares or adverse effects of immunosuppression, and maintenance therapy was maintained with routine outpatient follow-up.

## Conclusion

TKD is commonly associated with worse renal outcomes. Although rare, its presence in the isolated form in patients with SLE has been well described, and it is associated with progression to interstitial fibrosis and chronic kidney disease if it is not adequately treated. This case report reinforces the importance of understanding this relationship and seeking to diagnose it, with the objective of early therapeutic approach.

## Data availability statement

The original contributions presented in the study are included in the article/supplementary material, further inquiries can be directed to the corresponding author.

## Ethics statement

The studies involving human participants were reviewed and approved by Human Research Ethics Committee of the Federal University of Vale do São Francisco (Reference No. 83840317.4.0000.5196). The patients/participants provided their written informed consent to participate in this study. Written informed consent was obtained from the individual(s) for the publication of any potentially identifiable images or data included in this article.

## Author contributions

OG, LS, RD, GS, and PN acquired data, analyzed data, and wrote the original manuscript. MR, JN, DB, MM, and NS-F edited and revised the manuscript. All authors contributed to the article and approved the submitted version.

## Conflict of interest

The authors declare that the research was conducted in the absence of any commercial or financial relationships that could be construed as a potential conflict of interest.

## Publisher's note

All claims expressed in this article are solely those of the authors and do not necessarily represent those of their affiliated organizations, or those of the publisher, the editors and the reviewers. Any product that may be evaluated in this article, or claim that may be made by its manufacturer, is not guaranteed or endorsed by the publisher.
